# “Emotional stress is more detrimental than the virus itself”: A qualitative study to understand HIV testing and pre‐exposure prophylaxis (PrEP) use among internal migrant men in South Africa

**DOI:** 10.1002/jia2.26225

**Published:** 2024-03-10

**Authors:** Maria Francesca Nardell, Caroline Govathson‐Mandimika, Salomé Garnier, Ashley Watts, Dolapo Babalola, Nkosinathi Ngcobo, Lawrence Long, Mark N. Lurie, Jacqui Miot, Sophie Pascoe, Ingrid T. Katz

**Affiliations:** ^1^ Division of Global Health Equity Brigham and Women's Hospital Boston Massachusetts USA; ^2^ Harvard Medical School Boston Massachusetts USA; ^3^ Health Economics and Epidemiology Research Office (HE2RO) Johannesburg South Africa; ^4^ Faculty of Health Sciences University of the Witwatersrand Johannesburg South Africa; ^5^ EHESP French School of Public Health Paris France; ^6^ Harvard University Boston Massachusetts USA; ^7^ College of Medicine University of Ibadan Ibadan Nigeria; ^8^ Department of Global Health Boston University School of Public Health Boston Massachusetts USA; ^9^ Brown University School of Public Health Brown University Providence Rhode Island USA; ^10^ Division of Women's Health Brigham and Women's Hospital Boston Massachusetts USA

**Keywords:** men, migration, South Africa, HIV testing, HIV prevention, pre‐exposure prophylaxis

## Abstract

**Introduction:**

South Africa has one of the highest rates of internal migration on the continent, largely comprised of men seeking labour in urban centres. South African men who move within the country (internal migrants) are at higher risk than non‐migrant men of acquiring HIV yet are less likely to test or use pre‐exposure prophylaxis (PrEP). However, little is known about the mechanisms that link internal migration and challenges engaging in HIV services.

**Methods:**

We recruited 30 internal migrant men (born outside Gauteng Province) during August 2022 for in‐depth qualitative interviews at two sites in Johannesburg (Gauteng) where migrants may gather, a factories workplace and a homeless shelter. Interviewers used open‐ended questions, based in the Theory of Triadic Influence, to explore experiences and challenges with HIV testing and/or PrEP. A mixed deductive inductive content analytic approach was used to review data and explain why participants may or may not use these services.

**Results:**

Migrant men come to Johannesburg to find work, but unreliable income, daily stress and time constraints limit their availability to seek health services. While awareness of HIV testing is high, the fear of a positive diagnosis often overshadows the benefits. In addition, many men lack knowledge about the opportunity for PrEP should they test negative, though they express interest in the medication after learning about it. Additionally, these men struggle with adjusting to urban life, lack of social support and fear of potential stigma. Finally, the necessity to prioritize work combined with long wait times at clinics further restricts their access to HIV services. Despite these challenges, Johannesburg also presents opportunities for HIV services for migrant men, such as greater anonymity and availability of HIV information and services in the city as compared to their rural homes of origin.

**Conclusions:**

Bringing HIV services to migrant men at community sites may ease the burden of accessing these services. Including PrEP counselling and services alongside HIV testing may further encourage men to test, particularly if integrated into counselling for livelihood and coping strategies, as well as support for navigating health services in Johannesburg.

## 1 INTRODUCTION

Despite substantial progress, South Africa continues to have one of the world's largest HIV epidemics, with approximately 7.5 million people living with HIV [1]. While women shoulder a greater burden of HIV than men in South Africa [2], there are major gaps in reaching men with HIV services [3, 4, 5, 6]. In particular, only half of men living with HIV in South Africa know their status [3, 7], and levels of HIV testing for men remain consistently lower than for women [8, 9, 10]. Men with HIV also have lower rates of treatment adherence, viral suppression and survival [3, 11, 12]. Alongside efforts to help men living with HIV start and stay on treatment, it is imperative to focus on testing and prevention for HIV‐negative men [13]. Oral pre‐exposure prophylaxis (PrEP) is highly effective at preventing HIV [14, 15, 16, 17]. South African men have shown interest in using PrEP [18, 19], but barriers to men's use of HIV services include masculine norms, anticipated stigma, poverty and unemployment, and lack of knowledge about PrEP [3, 20]. In addition, most PrEP services in South Africa target women, men who have sex with men and sex workers [21, 22, 23, 24]. Concerns about the cost‐effectiveness of providing PrEP to heterosexual men may limit its implementation [25]. Despite this, South Africa's National Strategic Plan for HIV, TB, and STIs 2017–2022 recommends PrEP for anyone who reports being at risk for HIV, including heterosexual men [26]. Thus, it is important to understand how to target PrEP delivery to men for whom it may provide the greatest benefit.

South African men who move within the country, called internal migrants [27, 28], bear two to three times the burden of HIV as compared to non‐migrant men in South Africa [29, 30], and they also have a higher burden of HIV than international migrants [30]. Migrant men are at higher risk for HIV acquisition during the disruptive process of relocation [31] and engage in higher sexual risk behaviours [32]. Other research has shown that men living with HIV are more likely to become migrants than those who are HIV negative, in part due to marital dissolution, which people living with HIV are more likely to experience [27]. Compared to non‐migrants, internal migrants are less likely to use health services, more likely to use private services or traditional healers and less likely to test for HIV [33, 34]. Migrants are identified as a “key population” for HIV prevention [35], but only 15% of internal migrant men surveyed in community sites in Johannesburg were aware of PrEP [30]. HIV interventions that engage men in testing and prevention, such as workplace programmes and partner notification strategies [36], may exclude men who are unemployed or unpartnered, as is common for migrant men [30]. Moreover, large trials to improve HIV testing among men in South Africa have found substantial challenges reaching communities with high mobility and in‐migration [37]. South Africa has one of the highest rates of internal migration on the continent [38] with an estimated one million internal migrants settling in urban Gauteng Province from 2016 to 2021 alone, dwarfing the number of international immigrants by five to one [39].

Research to unpack the relationships between migration, masculinity, and barriers to HIV testing and prevention in South Africa has been limited. In particular, there is a lack of research on the ways in which HIV testing and prevention services are understood and experienced by internal migrant men, for whom xenophobia and lack of knowledge of the healthcare system may not apply [40, 41, 42]. There is also little research to understand how community‐based HIV delivery strategies may appeal to migrant men specifically [33, 43, 44]. Through this qualitative analysis of interviews with internal migrant men in Johannesburg, South Africa, we seek to fill these gaps by exploring psychosocial and structural factors that influence HIV testing and prevention uptake among internal migrants to inform possible intervention strategies. South Africa must engage this key population to meet its national goal of eliminating HIV as a public health threat with “nobody left behind” [45].

## 2 METHODS

### 2.1 Study setting and participants

This study is based in Johannesburg, the most populous city in South Africa. In our team's prior pilot work among migrant men in Johannesburg, we chose community‐based recruitment sites in the Hillbrow, Midrand, Woodmead and Roodeport areas following a period of observation and discussion with community stakeholders. For this study, we selected two sites with high proportions of internal migrant men. The first is an industrial area in Midrand, north of Johannesburg, where many migrant men work in factories. No healthcare services are delivered near this site. The second is a homeless shelter in Hillbrow (Johannesburg central), the Displaced Persons Unit (DPU) for men recently arrived in Johannesburg. We chose to focus on community sites in order to reach men who may not seek care at clinics. The DPU is adjacent to the public Hillbrow Community Health Care Centre, which provides HIV services and care, but men were not recruited directly from the clinic.

Recruitment occurred in August 2022 using convenience sampling. Team members recruited participants by asking leadership at the sites if the study team could recruit there on specific days. On site, the research assistants (RA) distributed study flyers. These flyers gave information about the study and invited men to contact the study team if they were interested in participating. Men who made contact were given further information and taken through the consent process in‐person. The team checked telephone numbers, names and identifiers to ensure that the same participant did not enrol twice.

### 2.2 Eligibility and sampling

We included men who were ≥18 years, self‐reported as an internal migrant (defined as a South African born outside of Gauteng Province), were willing to participate in a 45‐ to 60‐minute interview and were able to provide informed consent. Men were excluded from the study if they were believed to be intoxicated at the time of consent (in which case, they were invited to return the following day), unable to understand the study information or had previously enrolled in the study. In addition, we purposively sampled to seek perspectives from migrants who reported living in Johannesburg for a year or less, and those who lived in Johannesburg for longer durations.

### 2.3 Study design conceptual framework

Interviews followed an in‐depth semi‐structured interview guide developed to explore internal migrant men's experiences, knowledge and attitudes towards HIV testing and prevention. The development of our guide was informed by the Theory of Triadic Influence (TTI), a theoretical model of health behaviour used in several HIV‐related studies, including in South Africa and by our team previously [46, 47, 48]. TTI posits that there are three streams of influence on health‐seeking behaviour: *individual factors* (e.g. personal motivations, knowledge), *social factors* (e.g. interpersonal relationships, social norms) and *structural factors* (e.g. healthcare system structures and policies). Interview questions examined attitudes and behaviours towards HIV testing and PrEP within each of these streams of influence, such as knowledge of PrEP (*individual*), anticipated HIV stigma (*social*) and access to healthcare services (*structural*).

### 2.4 Data collection and preparation

Qualitative interviews were completed in person by one of three RAs in a private, secure setting in August 2022. (See Interview guide in online Appendix.) RAs were trained in qualitative interviewing and fluent in isiZulu, isiXhosa, English and seSotho; they conducted the interview in the participant's preferred language. Interviews lasted 45−60 minutes and were audio recorded. After completing the survey, participants were reimbursed for their time with a ZAR150 (∼USD10) electronic shopping voucher. All interview recordings were sent to a third‐party service for transcription and translation to English, if needed. Each interview was reviewed by the RA who conducted the interview to ensure accurate transcription and translation. Transcripts were assigned a unique study identification number to protect participant confidentiality.

### 2.5 Data analysis

The analysis aimed to characterize participants’ reasons for engaging or not with HIV testing and PrEP services. We used a mixed deductive and inductive approach informed by grounded theory [49]. Two team members (MFN and SG) developed an initial codebook based on key concepts from TTI and the interview protocol, as well as emergent codes from a review of the initial five interviews. The coding team (MFN, SG, DB and AW) discussed emerging themes through repeated review of the transcripts and memoing. The coding team further refined the codebook, resolving discrepancies through discussion, maintaining an audit trail of notes and each codebook version. Interviews were coded in Dedoose [50]. Each coding team member coded the first 20% of transcripts, and interrater reliability testing resulted in a pooled Cohen's kappa of 0.80. The four coders individually coded the remaining transcripts. We reviewed excerpts for each code, identifying repeated patterns of content which formed the basis for broader themes [49].

### 2.6 Ethical approval

The ethics committees at the University of the Witwatersrand (M191068), Mass General Brigham (Harvard University) (2020P002251) and Boston University (H‐40529) approved the study. All participants provided written informed consent.

## 3 RESULTS

### 3.1 Participant characteristics

We recruited 30 participants, half at the Midrand factories site and half at the Hillbrow homeless shelter. Demographic information for these participants is shown in Table 1. Nearly a third (30%) of participants were born in Limpopo Province, followed by nearly a fifth (16.7%) in KwaZulu‐Natal. Almost half (46.7%) of participants spoke isiZulu as their primary language and came to Johannesburg looking for work. Most reported no regular intimate or married partner (66.7%), though half (50%) had children. Nearly half (43.3%) reported living in Johannesburg for <3 years, and nearly a fourth (23.3%) for <6 months. Half (50.0%) of participants reported travelling to see family. Most participants had no regular form of employment. One participant reported living with HIV.

**Table 1:** Socio‐demographic characteristics of 30 participants

	Median	Range
**Age**	30	19−45

	**Number**	**Percentage**
**Site of recruitment**		
Factories	15	50.0
Shelter	15	50.0
**South African province of birth**		
Limpopo	9	30.0
Kwazulu‐Natal	5	16.7
Eastern Cape	4	13.3
Mpumalanga	4	13.3
Western Cape	2	6.7
Free State	2	6.7
North West	0	0
Northern Cape	0	0
No response	4	13.3
**Primary language**		
Zulu	14	46.7
Sisotho	10	33.3
Xhosa	4	13.3
Setswana	4	13.3
Sepedi	4	13.3
**Reason for coming to Johannesburg**		
Looking for work	14	46.7
Unspecified opportunity	7	23.3
Family reason	2	6.7
Problems in home of origin	2	6.7
No response	5	16.7
**Time in Johannesburg**		
<6 months	7	23.3
6 months–3 years	6	20.0
>3 years	10	33.3
No response	7	23.3
**Employment**		
Regular employment	3	10.0
No regular employment	23	76.7
No response	4	13.3
**Travel since being in Johannesburg**		
Travel to see family for holidays	6	20.0
Unspecified travel to see family	9	30.0
No reported travel	10	33.3
No response	11	36.7
**Partner**		
Yes	9	30.0
No	20	66.7
No response	1	3.3
**Children**		
Yes	15	50.0
No	13	43.3
No response	2	6.7
**Self‐reported HIV status**		
Negative or unknown	29	96.7
Positive	1	3.3

### 3.2 Qualitative results

We organized themes into barriers to and opportunities for care engagement, and we organized factors at individual, social and structural levels. Within these levels, we identified factors that apply to men more generally as well as factors specific to migrants.

### 3.3 Individual barriers

Participants framed their HIV testing decisions within the context of high levels of daily stress. As shown in Table 2, migrant men described a “weight […] that is really heavy from problems and life situations,” including “depressing thoughts” about finding work. Adding to their stress was awareness of HIV risk from condomless sexual encounters. For most, this risk awareness was not a motivator to seek HIV testing. Rather, HIV testing was more often perceived as an added burden because of fear that a positive result “might be too stressful for them.” While almost all participants were knowledgeable about antiretroviral therapy (ART) to manage HIV, they nonetheless described the possibility of a positive diagnosis in dire terms, including “about to die,” “ruin my life,” “like suicide,” “painful,” and “make [me] more sick.” As one participant described, “We as men are already carrying a lot. To add another weight on top of that… Emotional stress is more detrimental than the virus itself.” This burden was further exacerbated for many participants by virtue of their move to Johannesburg. Migrant men described challenges with navigating a new city, finding health services, and sometimes personal and family challenges that preceded their move to Johannesburg, which further taxed their cognitive and emotional bandwidth.

**Table 2:** Individual‐level barriers to HIV testing and PrEP use

Barriers	Examples
Coping with life stressors	“Naturally men have that weight over them that is really heavy from problems and life situations. […] Knowing that I have to test is going to put on more weight on me. I feel it will be adding to the problems to go and test. It is going to give me depression to find out that I am HIV positive. It is not that we do not want to test. It's just that we do not want to know at that certain time.” (Migrant from Western Cape, 11 months)
Fears of testing HIV positive	“People are scared to test. They will rather live in fear not knowing then to find out they are about to die.” (Limpopo, 4 months)
Lack awareness/knowledge of PrEP	“I do not know about [PrEP]. I have only heard rumours. I have never come across someone who have taken them.” (Mpumalanga, 3 months)
Seek healthcare for symptoms	“When I need healthcare I go to a public clinic because it is free. I wait and see if the sickness lasts for 48 hours then I go to the clinic. Anything that lasts for less than that I can handle.” (Mpumalanga, 3 months)

Complicating this narrative was limited awareness about PrEP for most participants. Those who had heard of it rarely knew anyone who had used it, where to find it or how to use it. Participants also mentioned low awareness of HIV prevention options in rural areas of the country, where many migrants had been raised. Given most participants did not know PrEP was an option if they tested negative, HIV testing was associated with few upsides. Fears of testing positive outweighed the potential for relief if they tested negative. While all participants reported testing for HIV at least once in their life, most tested only when required by a healthcare provider, rather than for prevention. Many reported seeking care only when they were ill or had lasting symptoms, as opposed to seeking care for regular or routine check‐ups. The interviewer's mention of PrEP was usually met with interest, with several respondents mentioning how they would trust PrEP if it came from healthcare professionals and after they “see it works for certain individuals.”

Only one study participant was living with HIV and reported testing five times to “be sure” that he was positive. Ultimately, he reported receiving HIV counselling and taking ART. He was knowledgeable about PrEP and regretted not having used it before acquiring HIV.

### 3.4 Social barriers

Gender norms played a large role in influencing participants’ beliefs and behaviours around HIV testing and PrEP. Participants described how HIV testing was a woman's responsibility, and a female partner's status was assumed to reflect their own. As shown in Table 3, most participants reported that clinics are designed to serve women's needs, and the predominance of female healthcare providers could be a disincentive to seeking care. Some migrants described how traditional gender norms were common in their rural homes of origin, and it was hard to shift this perspective in Johannesburg. Additionally, social gatherings with other men, particularly involving alcohol, emerged as a barrier to PrEP use. Participants described how men “are most vulnerable” when they drink because it distracts them from taking pills. Drinking also increases their HIV risk because men are less likely to use condoms when intoxicated.

**Table 3:** Social‐level barriers to HIV testing and PrEP use

Barriers	Examples
Social gender norms	“Women do not have such problems because the clinics are created for them. Women bear children. So already, they are used to the clinic. Women have many illnesses. I think it is women that transfer infections to men. Maybe going to a men's clinic would be better. It is quite a challenge to be seen by women going to the clinic.” (Western Cape, 3 months)
Social alcohol use	“It is really difficult to help men. Most men like to drink… Being drunk can make them miss the pill if it to be taken daily.” (KwaZulu‐Natal, unknown duration)
Anticipated stigma	
Testing HIV positive	“I think we worry that when we go to test, we will meet other people we know there, and we are concerned about what they would think.” (Mpumalanga, unknown duration)
PrEP	“People will mistake PrEP for ARV. They might even gossip about you saying you are using ARVs.” (Limpopo, 12 years)
Travel and migration	“Different people, different places. You must be prepared to be misunderstood [when traveling].” (Free State, 10 months)

Anticipated stigma was reported by nearly all men, both in clinics and in communities more broadly. One participant felt that being a newcomer in Johannesburg increased his risk of being “misunderstood” by people who did not know him. Although most participants reported being comfortable with others having HIV, they worried that testing positive would negatively affect their relationship with partners, family or friends, who “discourage each other from testing.” Mostly, the fear of stigma was non‐specific and related to the wider community: respondents reported that men “worry about what people would say” or “that they will bump into someone they know” at the clinic, in a general sense. PrEP use posed additional concerns, including the fear that PrEP would be mistaken for ART or signal infidelity. A few participants who knew of PrEP mentioned that PrEP is associated with men who have sex with men, which prompted some men “to shy away from using it.”

### 3.5 Structural barriers

Structural factors emerged as the largest barrier to HIV testing and PrEP use for participants. Finding and maintaining work was their biggest priority. Some had intermittent “piece jobs,” that is unpredictable day jobs in which men are paid by the hour or day. Others described how their jobs were unexpectedly terminated or how their pay was insufficient to afford housing or food. Given pressures to work, time was a major barrier to HIV testing, especially at clinics where participants reported queuing all day. Spending time at the clinic meant missing potential work opportunities. Many migrants reported coming to Johannesburg given its reputation as the “City of Gold” where “opportunities are broad.” For them, the gap between their dreams and reality was especially stark. Participants reported that work was not just a means of survival but the necessary precursor to “a home, wife, and kids…a dream [which] has not materialized.” On the other hand, for those participants with a family, the immediate need to provide for their children emerged as a higher priority than HIV testing.

On top of their livelihood concerns, newly arrived migrants described challenges adjusting to life in Johannesburg, including concerns about the fast pace of the city and their own safety. Participants described being unfamiliar with where HIV services are located, lacking support to access services or not trusting services from people they do not know. Migrants of any duration of time also reported that travel could increase HIV risk as they were more likely to have unprotected sexual encounters during these times. None of the participants used PrEP currently, though some hypothesized that travelling, including overnight travel for piece jobs, might pose a challenge for taking a daily pill (Table 4).

**Table 4:** Structural‐level barriers to HIV testing and PrEP use

Barriers	Examples
Employment opportunity costs	“I have to spend the entire day on the street by the robot waiting for an opportunity for piece‐work. If I go for testing I might miss that opportunity.” (KwaZulu‐Natal, unknown duration)
Travelling for work	“Because I am always on the go. Even with the piece‐jobs sometimes I don't sleep at home. I am concerned that I might miss a pill.” (Limpopo, 20 years)
Adjustment to Johannesburg	“I am still new in Johannesburg. It is a busy city. It is full of criminals. You cannot even ask people for directions to the clinic. Sometimes health facilities are located downtown where we cannot go because it is dangerous.” (Mpumalanga, 3 months)

### 3.6 Opportunities

As shown in Table 5, participants described several opportunities for engagement in HIV testing, prevention and care. Participants noted that HIV services in community‐based settings such as pharmacies, pop‐up tents and shops made access easier. Most appreciated the anonymity they felt seeking healthcare in Johannesburg as compared to rural areas. In fact, some participants reported that if diagnosed with HIV, they would feel most comfortable seeking support from healthcare staff in Johannesburg. More readily available HIV information in Johannesburg was also perceived as an opportunity for care engagement. As one participant shared, “I think people need to be informed about PrEP. This will encourage more people to do HIV testing so they can use PrEP if their results are negative.”

**Table 5:** Opportunities for HIV testing and PrEP use

Opportunities	Examples
**Differentiated service delivery**	“I would be comfortable accessing PrEP even outside the clinic, in pharmacies and shops. That will make it even more accessible to people.” (Eastern Cape, 15 years)
**Anonymity**	“Yes, I am comfortable [testing for HIV] in Johannesburg and free. There are no chances of meeting somebody I know.” (Limpopo, 10 months)
**PrEP information**	“PrEP need to be advertised more. It needs to get out there.” (KwaZulu‐Natal, 1 month)
**Social support**	“At the shelter there is tons of us. […] There is six of us in a room. They are my new family.” (Limpopo, 4 months)
**Masculinity**	“I sit down and talk to myself and tell myself that I am a man and that I can face any challenge.” (Limpopo, 7 years)
**Positive coping skills**	“In the past I was very scared to test. But I have matured and I always want to go and test for HIV. When I go I come back happy. I always expect anything and I have courage to deal with whatever results I get. I think I am comforted by the fact that HIV can be managed by treatment.” (KwaZulu‐Natal, unknown duration)

Social support from other men surfaced as an important resource for some participants, including through religious organizations, bars and sporting activities. At the homeless shelter, newly arrived participants described how men would gather to “share stories every night before we go to bed,” while others had support from family members or friends already in Johannesburg. Among these men, discussions about HIV were more prevalent than in their hometowns. One participant shared, “Here people are always talking about their HIV status. That pushes you to want to know yours.” In contrast to participants who described their sense of masculinity as a deterrent to care‐seeking, other men felt motivated by a sense of responsibility to their family: “You don't want to die and leave your family behind.” Others drew on their sense of masculinity or duty towards their partner: “I am the man and I have to protect my partner.” Lastly, some participants described positive coping skills which help them to feel “courage to deal with whatever results” from HIV testing.

## 4 DISCUSSION

**Figure 1:**
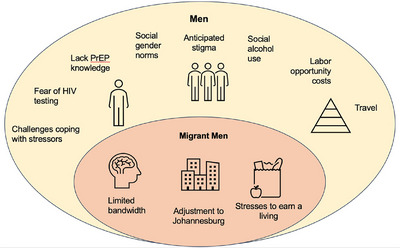
Barriers to HIV testing and prevention for men and internal migrant men in Johannesburg. In the yellow oval in the figure below, we show themes found to be common to men more generally, including migrants, organized by individual, social and structural levels of influence. In addition, we found themes which were heightened for migrant men, shown in the pink oval.

Our findings of barriers to and opportunities for HIV testing and PrEP in Johannesburg revealed themes common to men generally as well as themes exacerbated for or unique to internal migrant men across all levels of influence (individual, social, structural), as shown in Figure 1. Similar to prior research, we found that substantial fears of an HIV‐positive diagnosis and anticipated stigma remain major barriers to seeking HIV testing for men [47, 48, 49, 51, 52, 53, 54]. Building on this research, our data highlight that men worry that a positive HIV diagnosis on top of pre‐existing livelihood stressors would overwhelm their ability to cope. Unemployment rates in South Africa are greater than 30% [55], and studies have shown the stressful toll that unemployment takes on South African men, leading to financial strain, lower self‐esteem and decreased social status [56]. This suggests the need for interventions for men that address both livelihood stressors and coping skills. For example, a livelihood support intervention among persons living with HIV in Kenya showed evidence of mental health improvements [57], and the MenStar Strategy to engage men in HIV care in South Africa includes a focus on building coping skills [58]. However, problematic alcohol use among men—prevalent in resource‐constrained settings in South Africa and often the foundation of social gatherings [59]—further complicates this picture as alcohol use may be associated with HIV risk behaviours [60]. Efforts to address alcohol use and HIV risk together have had mixed success, with evidence showing that social barriers (e.g. social norms around substance use) and structural‐level barriers (e.g. unemployment, poverty, violence, limited clinic resources) constrain long‐lasting behaviour change [61, 62].

Building on these barriers for men, our data uncovered challenges that are exacerbated for and/or unique to internal migrants. The International Organization for Migration (IOM) recognizes that migrants have a higher likelihood of life stressors, given the conditions that prompted their move as well as their near‐universal need to find employment. In a report on the wellbeing of economic migrants in South Africa, the IOM lists safety concerns, lack of social support, poor access to healthcare, high unemployment, housing insecurity and insufficient access to food as major challenges characterizing migrant men in the country [63]. The migrant men in our study are similarly faced with numerous social and structural stressors by virtue of needing to start afresh, including securing housing, navigating a new health system, building a new social network, prioritizing physical safety and adjusting to the fast pace of life in Johannesburg. The experience of being new to a city may heighten the expectation of stigma among migrants, and this anticipated stigma from the broader community discourages many from seeking care, out of fear of being seen at the clinic or taking pills. Additionally, though about half of the participants mentioned coming to Johannesburg for work, only three reported regular employment. Each stressor takes up some degree of an individual's “cognitive bandwidth,” which encompasses both cognitive capacity (psychological mechanisms which allow for reasoning, problem‐solving and retaining information) and executive control (ability to plan, manage attention and control actions) [64]. Individuals have a finite amount of cognitive bandwidth. An accumulation of stressors can tax this bandwidth and impede cognitive functioning, making it more difficult to manage immediate stressors, let alone to process new information (e.g. about PrEP) or prioritize HIV testing or preventative care [64, 65, 66]. Similarly, research among international migrants has found that high levels of unemployment and competing life priorities, especially in the early phase of resettlement, can make it harder to seek preventative care [67].

Despite these challenges, our data identified opportunities to engage migrants in HIV care, particularly for new arrivals to Johannesburg. As in many countries, rural communities in South Africa often have less access to healthcare as compared to urban centres [68], and fears of unintentional disclosure may be higher in rural clinics [69]. While some research shows that HIV‐related stigma may be declining in South Africa [70], our data show that anticipated stigma nonetheless continues to be a concern for migrant men; thus, anonymity for new migrants is a preferred feature of care in Johannesburg. Johannesburg offers migrants greater opportunities to seek convenient, anonymous care, and also to learn about HIV prevention options, such as PrEP. Other research among migrants has also shown that many first learn about PrEP in their destination countries [67]. In addition, migration may lessen the influence of family and culture norms from one's home of origin, for example social constructs of gender which may limit men's care‐seeking [53]. While some of our participants reported social isolation, others described information sharing and new bonds that formed between themselves and other migrant men, especially in the shelter. Thus, there may be an opportunity to provide easily accessible HIV services to newcomers to Johannesburg at a time when they may be less constrained by prior social obligations and freer to adopt new social norms and behaviours [71]. Research has shown that community‐based service delivery for HIV testing and HIV treatment, including home and mobile van delivery, can help strengthen men's engagement with the care continuum, especially when combined with targeted messaging to motivate men [44, 72]. Similarly, in our study, many participants shared that accessing PrEP outside clinics (e.g. in pharmacies, pop‐up tents and shops) would help save time and increase anonymity, suggesting a role for scaling up these services. In particular, community pharmacies were appealing, as participants shared that pharmacists are considered both knowledgeable and trustworthy. This finding is consistent with other studies in sub‐Saharan Africa, which have shown that pharmacies are perceived as more convenient, more private and less stigmatizing than clinics, particularly by men [73, 74, 75]. Thus, our study supports the important role of community pharmacies in differentiated HIV care delivery for men in South Africa [76, 77] and in sub‐Saharan Africa more broadly [75, 78, 79].

Migrants have a right to healthcare within the South African constitution, including free HIV services through public healthcare providers [80, 81]. The on‐the‐ground reality of migrants’ challenges in accessing or even knowing about PrEP highlights the importance of addressing persistent community‐level barriers, such as using community health workers to help migrants navigate local health resources [82, 83]. In addition, South Africa's policy discourse tends to focus on the potential negative implications of migration and often obscures the needs of internal migrants [84]. Shifting this discourse at the national and local levels may help to generate the necessary public will to effectively respond to migration and health.

This study must be interpreted within the limitations of this work. Many participants in our sample were unaware of PrEP, and none reported using it, similar to other research on PrEP uptake for men in sub‐Saharan Africa [20, 85, 86]. Thus, PrEP may have theoretical appeal when first hearing about it, and social desirability bias may impact participants' enthusiasm for PrEP, while barriers to PrEP use may emerge more prominently for men with experience using it. In addition, given the nature of this qualitative study and our focus on two recruitment sites in Johannesburg, the results cannot be generalized to all migrant populations outside of Johannesburg, nor can we statistically analyse differences between different sub‐populations, for example newcomers versus longer‐term migrants. Rather, we aimed to shed light on mechanisms underlying engagement in HIV testing and prevention to deepen our understanding of experiences for a convenience sample of internal migrants seeking shelter and work in the city. Additionally, the men who were included in the study may not have been fully representative of the internal migrant population of Johannesburg, given that sampling relied on self‐selection into the study. Men who were approached but declined to participate may have differed from those included in terms of regular employment, family obligations or lack of interest in HIV prevention. Finally, many of the themes we uncovered were similar to those found previously for men more generally, suggesting that strategies to engage migrant men may have benefits for other men as well. Future research focusing on international migrants in South Africa is also warranted to better understand factors influencing their HIV care engagement.

## 5 CONCLUSIONS

Accessing HIV services in South Africa is enshrined in article 27(1)(a) on the right to health and to access healthcare services in the Constitution and Bill of Rights [87]. Migrant men in South Africa are considered a key vulnerable population and have been so for several decades, yet there is still limited awareness and information about PrEP among this population [88]. Migrant men come to Johannesburg to find work, but their struggle to survive without reliable income causes daily stress. Despite caring about their health, constraints on their time and cognitive bandwidth limit their ability to seek health services, particularly given fears of testing positive for HIV, anticipated stigma and limited knowledge about the opportunity for PrEP. Yet, Johannesburg also presents opportunities for HIV testing and PrEP use for migrant men who perceive greater availability and anonymity of HIV information and services in the city as compared to rural homes of origin, and also more willingness to adopt new beliefs and behaviours. Bringing HIV services to migrant men at community sites may ease the burden of accessing these services. Including PrEP counselling and services alongside HIV testing may further encourage men to test, particularly if integrated into counselling on livelihood and coping strategies, as well as support for navigating health services in Johannesburg.

## COMPETING INTERESTS

The authors have no competing interests to declare.

## AUTHORS’ CONTRIBUTIONS

MFN conceptualized the study design, with support from SP, ITK and CG‐M. NN conducted qualitative data collection, with supervision from CG‐M. MFN, SG, AW and DB coded qualitative data. MFN, SG, AW, DB, CG‐M and NN participated in qualitative data analysis, with guidance from SP, ITK, MNL, LL and JM. MFN drafted the initial manuscript. All authors reviewed the manuscript and supported with edits.

## FUNDING

This work has been made possible by the generous support of the American People and the President's Emergency Plan for AIDS Relief (PEPFAR) through the United States Agency for International Development (USAID) under the terms of Cooperative Agreement 72067419CA00004 to HE^2^RO. This work was supported by the Harvard Global Health Institute Burke Fellowship. MFN was supported by grant number T32 AI007433 from the National Institute of Allergy and Infectious Diseases (NIAID) and the Harvard University Center for AIDS Research (CFAR) NIH/NIAID fund under grant number 5P30AI060354‐18. LL was supported by the National Institute of Mental Health of the National Institutes of Health under grant number K01MH119923.

## DISCLAIMER

The contents are the responsibility of the authors and do not necessarily reflect the views of PEPFAR, USAID, the United States Government or National Institutes of Health.

## ETHICS APPROVAL AND CONSENT

The ethics committees at the University of the Witwatersrand (M191068), Mass General Brigham (Harvard University) (2020P002251) and Boston University (H‐40529) approved the study. All participants provided written informed consent.

## Supporting information


**Appendix**: Qualitative Instrument for Migrant Men's HIV Testing and Prevention, Johannesburg

## Data Availability

All study data and materials are available upon request by emailing the corresponding author.
