# Supplementary material for: “Emotional stress is more detrimental than the virus itself”: A qualitative study to understand HIV testing and pre‐exposure prophylaxis (PrEP) use among internal migrant men in South Africa
Source: J Int AIDS Soc. 2024 Mar 10;27(3):e26225. doi: 10.1002/jia2.26225 (PMC10935710; doi:10.1002/jia2.26225)
Supplement: Supplementary file 1 — Appendix: Qualitative Instrument for Migrant Men's HIV Testing and Prevention, Johannesburg [file JIA2-27-e26225-s001.docx]

**Appendix**

**Qualitative Instrument for Migrant Men’s HIV Testing and Prevention, Johannesburg**

**INTERVIEW**

**Socio-Demographic Information and Warm-up**

1. Can you tell me a bit about yourself? Do you work, and if so, what kind of work? How do you spend most of your time? Where do you spend most of your time? Where is your home located, and what is it like? Who lives with you? Do you have children? Do you have a wife or partner? When were you born? Where were you born? Which languages can you speak, and how would you describe your ethnicity? What is your highest level of schooling?
2. Can you tell me about your experience coming to stay in Johannesburg? Where did you live before Johannesburg? How long have you been coming here? Why did you decide to come here? Do you live in Johannesburg all the time or do you travel to other parts of South Africa? If you travel to other parts of the country, why do you go?
3. When you need healthcare services, where do you go and why? How do you decide when to go? When did you last visit a healthcare facility? Do you have medical aid?

**HIV Testing**

Now, I’d like to focus on questions related to HIV testing.

1. What kinds of things have you heard from people in your community about HIV?

- What have you heard from the government? From the media? Is religion important to you, and if so, what have you heard from religious organizations?
- What do you think of this information?

1. In general, what do you think are the major problems and challenges facing people who want to get tested for HIV? Are there particular challenges for men, and if so, what might those be? Are there particular challenges for people who are new to Johannesburg or travel frequently, and if so, what might those be?
   - Who should be responsible for HIV testing? Why do you think that?
2. What has been your experience getting tested for HIV? Where did you go? Was it within or outside of Johannesburg? What was your reason for choosing to test within or outside of Johannesburg? How were you treated? How often do you go?

- Do you know the results of your HIV testing, and if so, would you be willing to share the results with us, remembering that there is no way we can link what you tell us to your name?

****The rest of the interview is divided into two sections, one for participants who self-identify as HIV NEGATIVE OR UNDISCLOSED (see directly below), and one for participants who self-identify as HIV POSITIVE (see page 4). Please skip to the section that applies to this participant now.****

**If HIV NEGATIVE OR UNDISCLOSED:**

1. What have been some of the challenges that have affected whether or not you get an HIV test?

- Are these challenges you faced getting a test in Johannesburg or when you have been outside of Johannesburg? Are these challenges the same or different outside of Johannesburg, and why or why not? How comfortable do you feel getting HIV testing in Johannesburg, and why or why not?
- How do you think that traveling to other places (for example, traveling for work opportunities or for family reasons) has affected your decisions about HIV testing?
- Can you describe any challenges with work or seeking work that make it difficult to test? What are the costs associated with having to get a test, and do they affect your decision to test?

1. Can you describe how you manage to cope when things don’t go well in your life?
   - Where and how would you seek support if you found out that you were positive for HIV?
   - How does this affect your decision to get HIV testing?

**HIV Prevention/Pre-Exposure Prophylaxis (PrEP)**

Now I’d like to talk about HIV prevention.

1. What do you know about how to prevent HIV? What have you heard from people in your community about HIV prevention?
   - Who should be responsible for thinking about HIV prevention? Why do you think that?
2. Do you know about medications for HIV prevention, called Pre-Exposure Prophylaxis or PrEP? What do you know about them? What do you think about them? When do you think they should be taken? Who should take them? Have you ever taken them? Do you know where you could access them?
   - If you haven’t heard about PrEP, what would be helpful to you in thinking about whether you would consider using PrEP?
3. In general, what do you think are the major problems and challenges facing people who may think about using PrEP?

- Are there particular challenges for men to use PrEP, and if so, what might those be?
- Are there particular challenges for people who are new to Johannesburg or travel frequently, and if so, what might those be? Are these challenges the same or different outside of Johannesburg, and why or why not?

1. How comfortable would you feel accessing PrEP in Johannesburg? Why or why not?

- How do you think that traveling to other places (for example, traveling for work opportunities or for family reasons) has affected your decisions about using PrEP?
- Can you describe any challenges with work or seeking work that make it difficult to use PrEP? What are the costs associated with using PrEP, and do they affect your decision to use PrEP?

1. How would you describe your level of risk to get HIV? Can you describe things you would do to try to prevent getting HIV?
2. Now please imagine a situation in which you were planning to use PrEP. What do you think about accessing PrEP outside of the usual clinic setting?

- What might be some challenges with getting PrEP outside of a clinic? What might be good about doing this?
- Who do you think should give these medications if they were offered outside of a clinic? Where would you want to collect them from? Would you want a specific provider or is there someone you would trust more to give these medications?

Before we end, I wanted to ask you if there is anything else you think we should have talked about or asked about today? Is there anything else you want us to know or do you think there is anything else you can tell us today?

**IF HIV POSITIVE:**

**HIV Testing**

1. What have been some of the challenges that have affected whether or not you have gotten an HIV test in the past (before your HIV diagnosis)?

- Are these challenges you faced testing in Johannesburg or when you were outside of Johannesburg? Are these challenges the same or different outside of Johannesburg, and why or why not?
- How do you think that traveling to other places (for example, traveling for work opportunities or for family reasons) affected your decisions about HIV testing in the past?
- Can you describe any challenges with work or seeking work that made it difficult to test in the past? What are the costs associated with having to get a test, and did those ever affect your decision to test?

1. Can you describe how you manage to cope when things don’t go well in your life?
   - Did you seek support when you found out that you were positive for HIV? If so, where and how did you do so?
   - Did you test again for HIV after you found out that you were positive? If so, how many times, and why?
   - Did you start treatment or seek further care after you tested positive for HIV? If not, why not?

**HIV Prevention/Pre-Exposure Prophylaxis (PrEP)**

Now I’d like to talk about HIV prevention.

1. What do you know about how to prevent HIV? What have you heard from people in your community about HIV prevention?

- Who should be responsible for thinking about HIV prevention? Why do you think that?

1. Do you know about medications for HIV prevention, called Pre-Exposure Prophylaxis or PrEP? What do you know about them? What do you think about them? When do you think they should be taken? Who should take them? Did you ever take them before your diagnosis? If so, where did you access them?
2. In general, what do you think are the major problems and challenges facing people who may think about using PrEP?

- Are there particular challenges for men to use PrEP, and if so, what might those be?
- Are there particular challenges for people who are new to Johannesburg or travel frequently, and if so, what might those be? Are these challenges the same or different outside of Johannesburg, and why or why not?
- Are there challenges with work or seeking work that would make it difficult for men to use PrEP? What are the costs associated with using PrEP, and do you think that they affect whether men use PrEP?

1. Although I know you said you have been diagnosed with HIV, please try to imagine a situation in which you were planning to use PrEP. What do you think about accessing PrEP outside of the usual clinic setting?

- What might be some challenges with getting PrEP outside of a clinic? What might be good about doing this?
- Who do you think should give these medications if they were offered outside of a clinic? Where would you want to collect them from? Would you want a specific provider? Is there someone you would trust more to give these medications?

Before we end, I wanted to ask you if there is anything else you think we should have talked about or asked about today? Is there anything else you want us to know or do you think there is anything else you can tell us today?
